# Role of 5-HT_1A_ and 5-HT_7_ Receptors in Memory Regulation and the Importance of Their Coexpression: A Systematic Review

**DOI:** 10.3390/biom15060762

**Published:** 2025-05-26

**Authors:** Alfredo Briones-Aranda, Daniela Flores-Durán, Rodrigo Romero-Nava, Josselin Carolina Corzo-Gómez, Refugio Cruz-Trujillo, Floribert Toalá-Sepúlveda, Blanca E. Del-Río-Navarro, Fengyang Huang

**Affiliations:** 1 Pharmacology Laboratory, Faculty of Human Medicine, Autonomous University of Chiapas (UNACH), Tuxtla Gutiérrez 29000, Mexico; e180083@unach.mx; 2 Postgraduate Studies and Research Section, Higher School of Medicine, National Polytechnic Institute, Mexico City 11340, Mexico; dany.0219@hotmail.com (D.F.-D.); roloromer@gmail.com (R.R.-N.); 3 School of Chemical Sciences, Autonomous University of Chiapas (UNACH), Pan-American Highway Ocozocoautla-Cintalapa Km. 2.5, Ocozocoautla de Espinosa 29140, Mexicorefugio.cruz@unach.mx (R.C.-T.); 4 Allergy and Immunology Research Laboratory, Hospital Infantil de México Federico Gómez, Mexico City 06720, Mexico; berio@himfg.edu.mx; 5 Obesity and Asthma Research Laboratory, Hospital Infantil de México Federico Gómez, Mexico City 06720, Mexico

**Keywords:** agonist 5-HT_7_, antagonist 5-HT_7_, agonist 5-HT_1A_, antagonist 5-HT_1A_, memory, mice, learning, rats, cognition, coexpression, interplay

## Abstract

The 5-HT_1A_ and 5-HT_7_ receptors play a key role in regulating cognitive processes and have been widely linked to the pathophysiology of depression, anxiety, and schizophrenia—disorders often associated with memory impairment. Recently, interest has grown in understanding how the coexpression of these receptors contributes to cognitive decline. This review explores the individual roles of 5-HT_1A_ and 5-HT_7_ receptors, as well as their coexpression, in memory regulation. The heterodimerization of these receptors at both pre- and postsynaptic levels, along with their colocalization in serotonergic, glutamatergic, GABAergic, and dopaminergic neurons, adds to the complexity of this interaction and may help explain the paradoxical effects of selective serotonergic drugs (agonists and antagonists). These findings underscore the need for further research into the 5-HT_1A_ and 5-HT_7_ receptor relationship in cognitive decline through diverse approaches, including targeted gene silencing, electrophysiology, and cell culture studies.

## 1. Introduction

Serotonin, or 5-hydroxytryptamine (5-HT), is a biogenic monoamine that can function as both a neurotransmitter and a neuromodulator [1]. A family of 14 subtypes of 5-HT receptors (5-HTRs) has been identified, divided into seven families (5-HT_1_ to 5-HT_7_), most of which are coupled to G proteins, except for 5-HT_3_R, which is a cation channel that allows the entry of Na^+^ and Ca^2^^+^. The 5-HT_1_Rs (subtypes A–F) are coupled to Gi/o proteins, thereby promoting the inhibition of adenylyl cyclase (AC) and decreasing cAMP production. The 5-HT_2_Rs (A–C) are coupled to Gq/11 proteins, activate phospholipase C, and increase inositol 1,4,5-trisphosphate and diacylglycerol levels. Meanwhile, 5-HT_4_R, 5-HT_6_R, and 5-HT_7_R are coupled to Gs proteins, which enhance both AC activation and cAMP levels. All the 5-HTRs exhibit a widespread neuroanatomical distribution [2,3,4]. This broad distribution enables 5-HT to influence the regulation of various physiological processes, including temperature, respiration [5], cerebral vascular tone, appetite, circadian rhythm, and motor control [6]. Additionally, it can modulate behavioral processes [7] such as mood, perception, aggression, sexuality, attention, and complex cognitive functions such as learning and memory [8,9].

One of the main receptors studied in cognitive processes is the 5-HT_1A_ receptor (5-HT_1A_R). Previous research has shown that this receptor plays an important role in the regulation of development [10,11], neuronal plasticity, thus acting as a modulator of learning and memory [12]. The administration of both agonists and antagonists of the 5-HT_1A_R in previous studies has reported contrasting effects regarding the involvement of this receptor in memory.

On the other hand, the *5-HT_7_* receptor (*5-HT_7_R*) has also been associated with the regulation of cognitive processes. The *5-HT_7_R*, located in the hippocampus (HC), has been implicated in learning [13]. Similarly to what has been reported in various studies on the *5-HT_1A_R*, opposing results have also been described regarding the pro-cognitive role of the *5-HT_7_R* [14], including cognitive impairment or lack of effect [15] following the administration of the agonists and/or antagonists of the *5-HT_7_R* in rodents studied in cognitive models [13].

In this regard, it is important to highlight the complexity of serotonergic neurotransmission regulation involved in cognition, partly due to its interaction with other neurotransmission systems, as some 5-HTRs are expressed in both glutamatergic neurons and GABAergic interneurons [16] and in multiple brain areas, including the HC. As a result, 5-HT released at the postsynaptic neuron can induce either excitation or inhibition of these cells [17].

Although the mechanisms through which these 5-HTRs influence cognitive processes have not yet been fully elucidated, evidence suggests that both serotonin receptors can undergo oligomerization, including the formation of homodimers [18] and heterodimers [19] in most brain structures, which could modify the functionality of either receptor in their respective second messenger systems [20].

The interaction between these two receptors holds significant clinical relevance, as both serve as an important foundation for the treatment of depression, anxiety, and cognitive impairment associated with psychiatric disorders such as schizophrenia [20]. Given that the role of both receptors in memory is a complex and controversial topic, it is essential to update the information on recent research findings to enhance the understanding of their involvement in cognitive processes.

In light of this, this review will analyze the individual roles of *5-HT_1A_R* and *5-HT_7_R*, as well as their coexpression in memory regulation.

### 1.1. General Characteristics of the 5-HT_1A_R

#### 1.1.1. Genetic Aspects

This serotonergic receptor was the first subtype to be cloned in 1987 [21]. The protein product of this genomic clone retains all the characteristics of the *5-HT_1A_R* [22], is located on chromosome 5 in humans [21] and chromosome 13 in mice [23] and lacks introns in its coding sequence [24]. The mouse 5-HT_1A_R gene shares 94% sequence homology with the rat gene and up to 88% with the human gene. It encodes a protein of 422 amino acids, 89% of which are identical to those in the human receptor [24].

The promoter structure of this gene has now been fully characterized [25]. Additionally, several single-nucleotide polymorphisms have been identified, some of which are linked to psychiatric disorders such as depression, suicidal behavior, and schizophrenia [26].

#### 1.1.2. Molecular Structure

The 5-HT_1A_R is composed of seven transmembrane domains made up of hydrophobic amino acid sequences and belongs to the G protein-coupled receptor (GPCR) family [21]. It has an extracellular amino-terminal region and an intracellular carboxyl-terminal region. The extracellular domain of the 5-HT_1A_ receptor contains three glycosylation sites, while the intracellular loops have three potential phosphorylation sites—one in the second loop and two in the third loop. The short intracellular terminal region plays a key role in coupling the receptor to the G protein [27].

Heterotrimeric G proteins consist of three subunits: α, β, and γ. They are classified into four subgroups based on the structural and functional similarities of the Gα subunit. The β and γ subunits form a βγ complex, which remains intact unless denatured. The α subunit is responsible for the binding of guanine nucleotides.

#### 1.1.3. Molecular Signaling

It has been demonstrated that the signaling cascades activated by this receptor in the central nervous system (CNS) are primarily coupled to Gi/o proteins, leading to the inhibition of AC [28]. However, specific neuronal signaling pathways activated by this receptor have also been identified. Binding 5-HT to the 5-HT_1A_R reduces neuronal activity and firing rate by opening hyperpolarizing potassium (K^+^) channels and closing calcium (Ca^+^) channels [29].

Additionally, 5-HT_1A_R signaling involves the βγ complex, which activates phospholipase C (PLC) in undifferentiated cells of the HC and the dorsal raphe nuclei (DRN). In prefrontal cortex (PFC) neurons, this receptor enhances the activation of calcium/calmodulin-dependent protein kinase II (CAMKII), suggesting that this pathway also plays a role in receptor signaling. Likewise, other signaling pathways, such as those regulated by growth factors—including mitogen-activated protein kinase (ERK1/2) and the PI3K-AKT-GSK3β pathway—as well as certain ion channels, may also be linked to this receptor [30].

The 5-HT_1A_R appears to adjust it signaling repertoire depending on the type of neuron and its location in pre- and postsynaptic regions. Notably, presynaptic 5-HT_1A_Rs are prone to desensitization following prolonged exposure to increased 5-HT availability, whereas postsynaptic 5-HT_1A_Rs do not exhibit this characteristic [31]. Based on these described properties, the postsynaptic 5-HT_1A_R is considered the primary inhibitory receptor in the serotonergic system.

#### 1.1.4. Neuroanatomical Distribution

5-HT_1A_Rs are widely distributed throughout the CNS, both at presynaptic and postsynaptic levels. Studies using autoradiography and immunocytochemistry techniques have identified these receptors in the cell bodies and dendrites of 5-HT neurons in the DRN and the median raphe nucleus [16,17]. In these regions, 5-HT_1A_Rs function as somatodendritic autoreceptors, precisely regulating 5-HT neuronal activity [31,32,33].

At the postsynaptic level, a high density of heteroreceptors has been detected in limbic and cortical areas, including the lateral septum, cingulate cortex and entorhinal cortex, with particularly high expression in the HC and amygdala [17,34]. As postsynaptic heteroreceptors, 5-HT_1A_Rs can reduce neuronal excitability and firing rates. These receptors are expressed in the soma and dendrites of pyramidal and granular neurons in the HC, with higher concentrations in the dendritic spines of pyramidal neurons [16].

The differences in the localization of presynaptic and postsynaptic 5-HT_1A_Rs reflect their different roles in neuronal regulation. Presynaptic 5-HT_1A_ autoreceptors are directly involved in modulating 5-HT neuronal function [28]. A key regulatory mechanism of these autoreceptors is desensitization, which occurs through their internalization in the DRN. However, this process is not observed in 5-HT_1A_ heteroreceptors, which are found in regions such as the HC [35].

#### 1.1.5. Functions

There are several reasons why this receptor has been extensively studied in the CNS. It plays a key role in the regulation of several physiological functions [36], contributes significantly to neuronal development and synaptic plasticity [10], regulates learning and memory [12], and is implicated in the mechanisms underlying disorders such as depression [37], anxiety, suicidal behavior, and other psychiatric conditions [38].

It is important to highlight the increasing focus on the role of this receptor in memory. Recent studies have used serotonergic drugs to characterize its function in this cognitive process, as will be discussed later in this review.

#### 1.1.6. Pharmacology

From this perspective, 5-HT_1A_R agonists exert different effects depending on their localization. When somatodendritic autoreceptors are activated by 5-HT_1A_R agonists, they inhibit the serotonergic neurons in which they are located and reduce 5-HT release. In contrast, at postsynaptic receptors, such as those in the HC, agonists enhance 5-HT neurotransmission [31].

8-Hydroxy-2-(di-n-propylamino)tetralin (8-OH-DPAT) was the first full agonist developed [39] and it was later discovered to also act as a partial agonist of 5-HT_7_R. Despite this, it remains widely used to study the role of 5-HT_1A_R in cognitive function in rodents. In addition to 8-OH-DPAT, numerous selective, partial, and mixed-activity agonists have been synthesized. For example, lurasidone has been characterized as a 5-HT_1A_R agonist and a 5-HT_7_R antagonist. A general overview of available 5-HT_1A_R drugs is provided in Table 1.

### 1.2. General Characteristics of the 5-HT_7_ Receptor

#### 1.2.1. Genetic Aspects

The 5-HT_7_R was cloned and characterized independently by researchers from several laboratories almost simultaneously in 1993 [40,41,42]. The 5-HT_7_R gene is located on human chromosomes *10q23.3-q24.3* and contains an open reading frame (ORF) of 1335 base pairs, encoding a 445-amino acid protein [40]. Unlike 5-HT_1A_R, this gene contains introns that interrupt its coding sequence, leading to the generation of multiple functional splice variants. Among these, the 5-HT_7A_ isoform is the most predominant (the first splice variant cloned in humans, with a predicted length of 445 amino acids), followed by the 5-HT_7B_ splice variant [43], both of which are expressed in humans and rats.

#### 1.2.2. Molecular Structure

The 5-HT_7_R also belongs to the large GPCR family, sharing the seven hydrophobic amino acid domains that characterize this superfamily of G protein-coupled receptors [42]. The N-terminal end is located on the extracellular side of the cell, while the C-terminal end is on the intracellular side. Residues within the transmembrane domains bind to ligands, while the intracellular domains interact with various cytoplasmic proteins to initiate downstream signaling pathways [44]. The human 5-HT_7_R shares 39–53% homology with the human 5-HT_1A_, 5-HT_2_, 5-HT_5_, and 5-HT_6_ receptors [40].

#### 1.2.3. Molecular Signaling

This receptor is coupled to Gs proteins, leading to an increase in AC activity, which in turn stimulates the production of intracellular cyclic adenosine monophosphate (cAMP). This cascade activates protein kinase A (PKA), inducing the phosphorylation of various target proteins and triggering multiple signaling pathways, including both Ras-dependent and Rap1-independent activation of the ERK and Akt neuroprotective pathways [45,46].

Additionally, 5-HT_7_R interacts with G_12_ proteins, which activate multiple signaling pathways. Among their key downstream effectors are small GTPases from the Rho family—Cdc42, RhoA, and Rac [47]. These molecules play a crucial role in regulating cell morphology, actin cytoskeleton, dynamics neurite branching, dendritic arborization, neurite outgrowth, synaptogenesis, and neuronal excitability [48]. Specifically, RhoA and Cdc42 promote cell-rounding and filopodia formation [47], processes involved in neuronal plasticity in various brain regions associated with memory formation and consolidation.

#### 1.2.4. Neuroanatomical Distribution

The 5-HT_7_R is widely expressed in the CNS, including the spinal cord [49]. Immunohistochemical analysis and in situ hybridization studies reveal high 5-HT_7_R expression in limbic structures, the thalamus, the hypothalamus (specifically the suprachiasmatic nucleus), the HC, and the amygdala [41]. Additionally, in the PFC, 5-HT_7_R is expressed in both neurons and glial cells [50].

Like 5-HT_1A_Rs, 5-HT_7_Rs are also found in the DRN in both rodent and human brains. At the neuronal level, 5-HT_7_R is expressed in the cell bodies of pyramidal neurons, dopaminergic neurons, and interneurons of the HC [51,52].

#### 1.2.5. Functions

In peripheral tissues, *5-HT_7_R mRNA* is present in the gastrointestinal tract, spleen, endocrine glands, genitourinary tract, and blood vessels. The expression of 5-HT_7_R in smooth muscle cells has been linked to the regulation of urination [42], direct vasorelaxation in multiple vascular beds [53], intestinal muscle relaxation [54], and peristaltic movement [55], among other functions [56].

In the CNS, 5-HT_7_Rs promote the formation of dendritic spines and protrusions and accelerate synaptogenesis in the HC, leading to increased spontaneous synaptic activity [48]. They also stimulate neurite outgrowth, particularly in striatal and cortical neurons [57]. Among other physiological responses, 5-HT_7_Rs are involved in the regulation of sleep [40], circadian rhythm [46], body temperature [47], and neuroendocrine regulation [48]. These receptors play a crucial role in behavioral and cognitive functions, particularly memory.

Previous experimental studies have supported the involvement of 5-HT_7_R in contextual learning and HC-dependent memory processing [15,58]. Furthermore, substantial evidence suggests that 5-HT_7_R is implicated in behavioral disorders such as anxiety and depression [49]. Several antipsychotic and antidepressant drugs with high affinity for 5-HT_7_R have also demonstrated pro-cognitive effects [40,50,59].

#### 1.2.6. Pharmacology

Over the past decade, various 5-HT_7_R agonists and antagonists have been developed to investigate their functionality in cognitive processes. Analyses have revealed that some antagonists induce irreversible inhibition of 5-HT_7_R [60], while others are potent but reversible antagonists that can be eliminated. Currently, the specific 5-HT_7_R antagonist 3-[(2R)-2-[2-(4-methylpiperidin-1-yl)ethyl]pyrrolidin-1-yl]sulfonylphenol (SB-269970) is widely used in both in vitro and in vivo studies [48,61].

Recently, novel selective agonists have been developed, including (2S)-N,N-dimethyl-5-(1,3,5-trimethylpyrazol-4-yl)-1,2,3,4-tetrahydronaphthalen-2-amine (AS-19), N-[(4-cyanophenyl)methyl]-6-[4-(2-phenylphenyl)piperazin-1-yl]hexanamide (LP-211), and 3-(2-aminoethyl)-1H-indole-5-carboxamide (5-CT) [62]. These serotonergic drugs serve as valuable tools for further exploring the role of 5-HT_7_R in cognitive processes.

Notably, new 5-HT_7_R agonists such as AS-19 and LP-211, as well as antagonists like lurasidone and SB-269970, exhibit high affinity for 5-HT_7_R. The specific affinity values of these compounds are detailed in Table 1.

**Table 1:** Affinity of 5-HT_1A_ and 5-HT_7_ serotonergic drugs.

**5-HT_1A_ Serotoninergic Drugs**			
**Agonists**	***Ki* (nM)**	**Antagonists**	***Ki* (nM)**
NLX-101 [63]	8.7	WAY-100635 [64]	0.24
8-OH-DPAT [65]	0.58	NAD-299 [64]	0.59
Lurasidone [66]	6.4	NAN-190 [65]	0.97
Flesinoxan [65]	0.54	WAY-100135 [65]	17
**5-HT_7_ Serotoninergic Drugs**			
**Agonists**	***Ki* (nM)**	**Antagonists**	***Ki* (nM)**
LP-12 [67]	0.13	Lurasidone [66]	0.5
LP-44 [67]	0.22	SB-258719 [68]	31.6
AS-19 [69]	2.5	SB-269970 [70]	1.3
LP-211 [71]	15	Vortioxetine [72]	19

The table provides a comparative overview of the affinity (*Ki*) of the main drugs for 5-HT_1A_R and 5-HT_7_R, ordered from highest to lowest affinity.

### 1.3. Oligomerization of 5-HT_1A_R and 5-HT_7_R

Previously, GPCRs were thought to exist and function as monomeric units that interact with their corresponding G proteins in a 1:1 stoichiometry. However, new evidence has demonstrated the ability of GPCRs to form oligomers [73,74]. Oligomerization can occur between identical receptor types (homodimerization) or between different receptors within the same family or from distinct GPCR families (heterodimerization). This process enables allosteric interactions between receptors, leading to a novel dynamic in which new allosteric binding sites may emerge [75,76], altering receptor recognition, signaling, and pharmacological effects. It is now widely accepted that homo- and heterodimerization may serve as an additional mechanism regulating GPCR-mediated signaling in the CNS [77]. GPCR heteroreceptor complexes can also involve ion channels and/or neurotransmitter transporters.

An additional level of complexity has been reported in 5-HT_1A_R signaling, including the possible formation of homodimers, heterodimers, and trimers [19], which are influenced by the presence of selective ligands that may induce changes in the density of this coexpression. Pharmacological analyses combined with BRET [18] and FRET [19] experiments have demonstrated that 5-HT_7_R can also form homodimers. Similarly, combined biochemical and biophysical approaches have shown that 5-HT_7_R can form heterodimers with 5-HT_1A_R both in vitro and in vivo [19]. However, the 5-HT_1A_/5-HT_7_ complex may have functional consequences, as heterodimerization reduces the G protein coupling of 5-HT_1A_R and attenuates its receptor-mediated activation of G protein-gated inwardly rectifying K^+^ (GIRK) channels, without substantial changes in 5-HT_7_R coupling to G_s_ proteins. Additionally, heterodimerization significantly facilitated 5-HT_1A_R internalization in the HC, while the internalization kinetics of 5-HT_7_R slowed following heterodimerization [19].

## 2. Methods

### 2.1. Literature Review and Information Selection Process

To construct and develop this review document, a qualitative systematic literature review was carried out following PRISMA guidelines [78]. The information was gathered from two databases: NCBI and Google Scholar. A set of keywords was used to identify relevant literature, including agonist 5-HT_1A_, 5-HT_7_, antagonist 5-HT_1A_, antagonist 5-HT_7_, localization, oligomerization, memory, learning, mouse, rat, and receptor. The guiding research question was “What role do 5-HT_1A_ and 5-HT_7_ receptors play in memory regulation?”. To ensure consistency, the selected articles were categorized based on key variables and their respective fields of study.

The inclusion criteria focused on in vitro and in vivo preclinical studies using animal models, published between 2012 and 2024, with an emphasis on the role of 5-HT_1A_, 5-HT_7_R, and their oligomerization in memory processes. The goal was to identify studies that fit the following criteria:

1. Reviewed previous neuropharmacological research where serotonergic agonists and antagonists (5-HT_1A_R and 5-HT_7_R) were administered to assess memory using different cognitive models.
2. Analyzed 5-HT_1A_R and 5-HT_7_R oligomerization on memory to integrate a theory that could correlate 5-HT_1A_/5HT_7_ heterodimerization with the pathophysiology of cognitive impairment.

Initially, studies were excluded if they were published in languages other than English or Spanish, focused on unrelated topics, lacked full-text availability, or did not include the specified keywords in the abstract or main text. During the screening process, records that were off-topic or whose study results were inconclusive or led to tentative conclusions were excluded. The review protocol is now available in the International Register of Systematic Review Protocols (CRD420251007975).

### 2.2. Data Extraction

Potentially relevant articles were identified and downloaded, followed by a keyword search. All duplicates were manually removed. The remaining reports were carefully reviewed to select those most relevant to the research question. The analysis was conducted using an Excel spreadsheet to extract key data, including study design, hypothesis, rodent characteristics, results, and main findings. The extracted details were rigorously compared across studies, given the limited available evidence, particularly regarding cognitive effects.

### 2.3. Risk of Bias Assessment in Selected Studies

The search for relevant reports and the selection process were conducted independently by two experienced reviewers with different areas of expertise. The risk of bias in preclinical experimental studies was evaluated using the RoB tool from the Systematic Review Centre for Laboratory Animal Experimentation (SYRCLE) [79]. Any disagreements between the reviewers were resolved through consensus-driven discussions or, if necessary, by consulting a third expert.

## 3. Results

### 3.1. Selection of Articles and Descriptive Data Analysis

**Figure 1:**
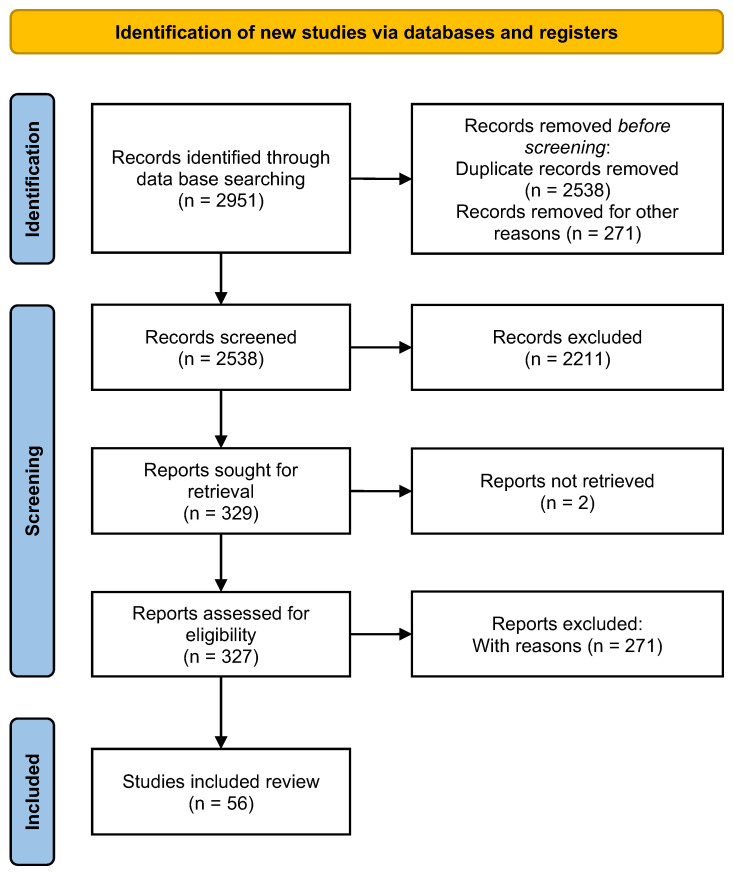
Literature review of serotonergic drugs (5-HT_1A_ and 5-HT_7_) and their coexpression associated with memory. The flow diagram illustrates the article selection process for this systematic review.

A total of 2951 potentially relevant publications were identified across two databases. Of these, 413 were duplicates, and 2482 did not meet the inclusion criteria. After a preliminary screening of the remaining 327 articles, 56 (with and without significant differences between groups) were selected for full-text review. The selection process is illustrated in the PRISMA flow diagram (Figure 1). The findings from the selected studies are summarized in relation to three areas of knowledge: information on 5-HT_1A_R agonists/antagonists (Table 2 and Table 3) and the effects of 5-HT_7_R agonists/antagonists (Table 4 and Table 5). Most of the reports considered in this review presented a low risk of bias.

### 3.2. Effects of 5-HT_1A_R Agonists on Memory

Table 2 shows that most previous studies have demonstrated a relationship between various 5-HT_1A_R agonists, such as 8-OH-DPAT [80,81], Flesinoxan [82], and (3-chloro-4-fluorophenyl)-[4-fluoro-4-[[(5-methylpyrimidin-2-yl)methylamino]methyl]piperidin-1-yl]methanone (NLX-101) [83], and procognitive effects in rodents. These effects were assessed using various behavioral models, including the Novel Object Recognition Test (NORT), Carousel Maze (CM), Conditioned Stimulus–Unconditioned Stimulus (CS-US) tested in the Passive Avoidance Task (PA), and Object Pattern Separation (OPS).

In contrast, some studies reported cognitive impairment or no effects following the administration of the 8-OH-DPAT agonist in rodents, particularly when assessed using the NORT [84,85].

**Table 2:** Effects of 5-HT_1A_R agonists on cognition.

References	5-HT_1A_ Agonist	Animals; Other Previous Treatments *(Dose of the 5-HT_1A_ Agonist)*	Behavioral Model *Main Effect*
Nikolaus et al., 2024 [85]	8-OH-DPAT	Rats ♂ (*3 mg/kg; i.p.—15 min*)	NORT *Cognitive impairment*
Wang et al., 2020 [84].		Mice ♂; β-Amyloid protein (*1 mg/kg; i.p.—1 h*)	-
Janikova et al., 2021 [86].		Rats ♂ (0.25 mg/kg; sc. During habituation)	CM -
Solís-Guillén et al., 2021 [80].		Rats ♂ (*0.3–0.62 mg/kg; i.p.—30 min*)	CS-US *Procognitive*
Pittalà et al., 2015 [81].		Mice ♂ (*1 mg/kg; s.c.—15 min*)	PA *Procognitive*
du Jardin et al., 2014 [82].	Flesinoxan	Rats ♂; PCPA (*1 mg/kg; i.p.—1 h*)	NORT *Procognitive*
van Hagen et al., 2022 [83].	NLX-101	Rats ♂ (*0.08 mg/kg; i.p.—30 min acutely, 0.32 mg/kg/day chronically × 14 days*)	OPS *Procognitive*

The table compares the effects of different doses of 5-HT_1A_R agonists on memory using various behavioral models. The terms and abbreviations used include the following: 8-OH-DPAT—8-Hydroxy-2-(di-n-propylamino)tetralin; PCPA—4-chloro-DL-phenylalanine methyl ester HCl; NORT—Novel Object Recognition Test; CM—Carousel Maze; CS-US—Conditioned Stimulus–Unconditioned Stimulus; PA—tested in the Passive Avoidance Task; OPS—Object Pattern Separation; no effect (-).

### 3.3. Effects of 5-HT_1A_R Antagonists on Memory

Contrasting effects have been reported following the administration of various 5-HT_1A_R antagonists, as shown in Table 3. Antagonists such as N-[2-[4-(2-methoxyphenyl)piperazin-1-yl]ethyl]-N-pyridin-2-ylcyclohexanecarboxamide (WAY-100635) [82,84] and (3R)-3-[di(cyclobutyl)amino]-8-fluoro-3,4-dihydro-2H-chromene-5-carboxamide (NAD-299) [87,88] have been associated with procognitive effects in rodents, assessed through different behavioral models, including the NORT and Water Maze. However, cognitive impairment or lack of effect has been observed in studies where the antagonist WAY-100635 was administered to rats evaluated in the NORT [89] or OPS [90].

**Table 3:** Effects of 5-HT_1A_R antagonists on cognition.

References	5-HT_1A_ Antagonist	Animals; Other Previous Treatments *(Dose of the 5-HT_1A_ Antagonist)*	Behavioral Model *Main Effect*
Wang et al., 2020 [84].	WAY-100635	Mice ♂; β-Amyloid protein (*0.5 mg/kg*; *ip—1 h*)	NORT *Procognitive*
Huang et al., 2018 [89].		Mice ♂; PCP (*0.6 mg/kg*; *i.p.—1 h*)	-
Afshar et al., 2018 [87].	NAD-299	Rats ♂; STZ (*5 μg/0.5 μL*; *icv*)	*Procognitive*
Gharib et al., 2019 [88].		Rats ♂; low frequency stimulation (LFS) (*5 μg/μL*; *intrahipocampal*)	Water maze *Procognitive*
van Goethem et al., 2015 [90].	WAY-100635	Rats ♂; F13714 *5-HT_1A_* agonist (*0.63 mg/kg; s.c.—1 h*)	OPS *Cognitive deterioration*
Solís et al., 2021 [80].		Rats ♂ (*0.3 and 0.6 mg/kg*; *i.p.—30 min*)	CS-US *Procognitive*

The table compares the impact of different doses of 5-HT_1_AR antagonists on memory. The terms and abbreviations used include the following: PCP—phencyclidine; STZ—streptozotocin; NORT—Novel Object Recognition Test; OPS—Object Pattern Separation; CS-US—Conditioned Stimulus–Unconditioned Stimulus; no effect (-).

### 3.4. Effects of 5-HT_7_R Agonists on Memory

Since 5-HT_7_R is one of the most recently discovered pharmacological targets, it remains one of the least characterized serotonergic receptors. As a result, information from previous studies on its functionality is limited, making its role more difficult to understand. This challenge is further compounded by the lack of selective 5-HT_7_R agonists, which poses a significant limitation in studying its role in learning and memory regulation. Additionally, the contrasting effects observed with 5-HT_7_R drug administration on memory add further complexity to the analysis.

For example, recent studies summarized in Table 4 indicate that the administration of 5-HT_7_R agonists in rodents, assessed using behavioral models such as the NORT [72,89] and CS-US [80,91], resulted in either no effect or procognitive outcomes.

**Table 4:** Effects of 5-HT_7_R agonists on cognition.

References	5-HT_7_ Agonist	Animals; Other Previous Treatments *(Dose of the 5-HT_7_ Agonist)*	Cognitive Model *Main Effect*
Huang et al., 2018 [89].	AS-19	Mice ♂ (*10 mg/kg*; *i.p.—30 min*)	NORT -
Westrich et al., 2015 [72].		Rats ♂ (*5 mg/kg*; *—4 h and—1 h*)	*Procognitive*
Solís et al., 2021 [80].	LP-211	Rats ♂ (*5–10 mg/kg*; *i.p. after last session*)	CS-US
Meneses et al., 2015 [91].		Rats ♂ (*0.5–1 mg/kg*; *i.p. after last session*)	*Procognitive*
		Rats ♂ scopolamine (*1 mg/kg*; *i.p. after last session*)	-

The table presents a comparative analysis of the behavioral effects observed with different doses of *5-HT_7_R* agonists. The terms and abbreviations used include the following: NORT—Novel Object Recognition Test; CS-US—Conditioned Stimulus–Unconditioned Stimulus; no effect (-).

### 3.5. Effects of 5-HT_7_R Antagonists on Memory

Similarly, contrasting effects were observed following the administration of various 5-HT_7_R antagonists, as detailed in Table 5. Antagonists such as vortioxetine [72,92] and lurasidone [89] were associated with procognitive effects in rodents when evaluated using the NORT behavioral model. However, another study reported that it had no effect when the selective antagonist SB-269970 was used in animals assessed with the CS-US model [80].

Additionally, further research involving SB-269970 revealed even more contrasting results. In some cases, a procognitive effect was observed in animals evaluated using the NORT model. However, a null effect was also reported in rodents assessed with the same behavioral model following the injection of a combination of SB-269970 and the 5-HT_7_R agonist AS-19 [72].

**Table 5:** Effects of 5-HT_7_R antagonists on cognition.

References	5-HT_7_ Antagonist	Animals; Other Previous Treatments *(Diagram of the 5-HT_7_ Agonist)*	Cognitive Model *Main Effect*
Solís et al., 2021 [80].	SB-269970	Rats ♂ (*10 mg/kg*; *sc. Immediately after*)	CS-US -
Liu et al., 2022 [93].		Mice ♂; isoflurane (*1 mg/kg*; *i.p.—3 days*)	NORT
Westrich et al., 2015 [72].		Rats ♂ (*4 mg/kg*; *i.p.—24 h and—1 h*)	*Procognitive*
		Rats ♂; AS-19 (*4 mg/kg*; *i.p.—24 h and—1 h*)	-
	Vortioxetine	Mice ♂ (*10 mg/kg*; *i.p.—24 h and—1 h*)	
Jensen et al., 2014 [92].		Rats ♀; PCPA (*10 mg/kg; i.p.—1 h*)	*Procognitive*
Huang et al., 2018 [89].	Lurasidone	Mice ♂; PCP (*0.3 mg/kg*; *i.p.—30 min*)	

The table compares the behavioral effects observed with different doses of 5-HT_7_R antagonists. The terms and abbreviations used include the following: PCPA—4-chloro-DL-phenylalanine methyl ester HCl; PCP—phencyclidine; AS-19—(2S)-(+)-5-(1,3,5-trimethylpyrazol-4-yl)-2-(dimethylamino)tetralin; NORT—Novel Object Recognition Test; OPS—Object Pattern Separation; CS-US—Conditioned Stimulus–Unconditioned Stimulus; no effect (-).

## 4. Discussion

Before analyzing and proposing a theory to explain the role of 5-HT_1A_R and 5-HT_7_R in cognitive impairment, several key premises must be revisited, which may help improve our understanding of this complex system.

First, cognitive processes are dynamic, as multiple mechanisms occur during different phases of memory formation. These mechanisms involve changes in neurotransmitter metabolism and reuptake, as well as modifications in the synthesis and coexpression of membrane receptors where these neurotransmitters act.

Second, it is crucial to consider the pre- and postsynaptic localization of both receptors (5-HT_1A_ and 5-HT_7_), along with their distribution across different neuronal cell types, including serotonergic, dopaminergic, glutamatergic, and GABAergic neurons. These receptors are present in brain regions involved in memory formation, such as the HC, striatum, and PFC [35,51].

Moreover, previous research findings, as described below, may provide further insight into the interaction of these factors and their impact on memory formation and consolidation, and example being differences in functionality between a 5-HT_1A_ autoreceptor and heteroreceptor [16,28,32].

It has been theorized that the activation level of 5-HT_1A_ autoreceptors may regulate postsynaptic 5-HT release, with high activation leading to low 5-HT release and low activation resulting in increased release [94]. This phenomenon is similar to the stimulation of serotonergic receptors that increase dopamine (DA) and glutamate (Glu) release in the HC. Additionally, another factor contributing to the complexity of the serotonergic system is the post-activation response of the 5-HT_1A_R, which can have paradoxical effects, either decreasing [28] or increasing cAMP synthesis. The latter effect is particularly observed in the presence of type II AC in the HC and NDR [30].

On the other hand, activation of the 5-HT_7_R, which is coupled to Gs proteins, increases cAMP levels, whether located pre- or postsynaptically [45,46]. It has also been described that in enterocytes, 5-HT_7_R activation enhances the functionality of the serotonin transporter (SERT), increasing 5-HT reuptake, while 5-HT_1A_R stimulation has the opposite effect [95]. A similar opposite effect has been observed for 5-HT_1A_R and 5-HT_7_R located on GABAergic interneurons, where stimulation of 5-HT_7_R is associated with increased gamma-aminobutyric acid (GABA) release, whereas activation of 5-HT_1A_R leads to a decrease in GABA release [89,96].

**Figure 2:**
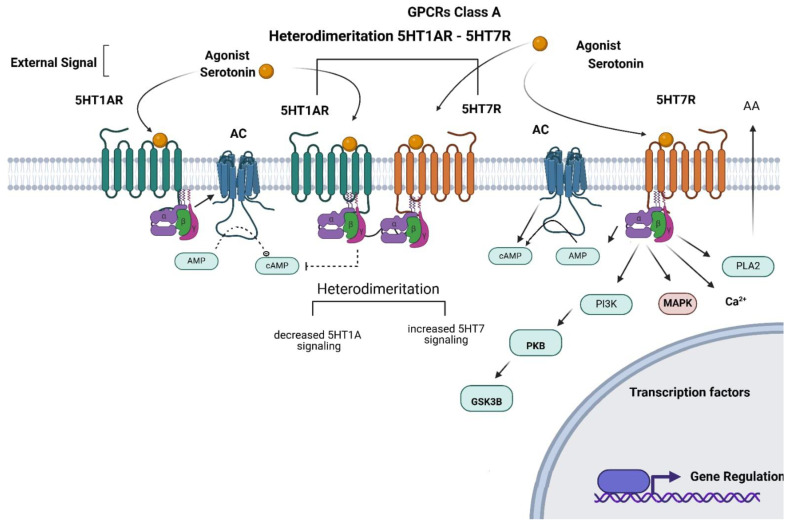
Activation of A class G proteins by 5-HT agonism in neuronal cells. (Left) 5-HT_1A_ activation by G_I_/_O_ signaling by decreasing cAMP levels. (Right) 5HT_7_ activation by G_S_ signaling by increasing cAMP formation and stimulating PLA2, Ca, MAPK, and PI3K. (Center) heterodimerization between 5HT_1A_ and 5HT_7_ favoring G_S_ signaling and decreasing G_I_/_O_ signaling. The terms and abbreviations used include the following: cAMP—cyclic adenosine monophosphate; PKA—protein kinase A; CREB—cAMP response element-binding; PLA2—phospholipase A2; MAPK—mitogen-activated protein kinases; PI3K—Phosphoinositide 3-kinase; PKB—protein kinase B; GSK-3β—Glycogen synthase kinase 3β. (Created in https://BioRender.com).

Another important biochemical mechanism gaining attention is receptor oligomerization, due to the functional changes induced by receptor dimerization [19]. For instance, an increase in 5-HT_1A_/5-HT_7_ heterodimerization has been shown to reduce 5-HT_1A_R functionality [19], potentially promoting its phosphorylation (Figure 2) and internalization [19,97]. In contrast, homodimerization of either 5-HT_1A_R or 5-HT_7_R does not appear to alter cAMP signaling upon activation.

Another important factor to consider in previous research is the influence of developmental progression on receptor density in mice. As rodents age, 5-HT_1_AR density appears to increase compared to 5-HT_7_R density in the HC and PFC [19]. This is consistent with findings from other studies showing that 5-HT_7_R expression in the HC progressively decreases during postnatal development [48].

Based on this, it has been theorized that under non-pathological conditions, the number of 5-HT_1A_R/5-HT_7_R heterodimers is greater in presynaptic serotonergic neurons than in postsynaptic neurons. However, in the postsynaptic region, there may be a balance between the amounts of heterodimers and homodimers [97]. This balance could contribute to maintaining a moderate and consistent tone in the postsynaptic release of key neurotransmitters involved in neuronal plasticity, such as 5-HT, DA, acetylcholine (ACh), and Glu.

In major depression, which can be associated with cognitive impairment [98,99], it has been proposed that the balance between 5-HT_1A_R/5-HT_1A_R homodimers and 5-HT_1A_R/5-HT_7_R heterodimers in presynaptic serotonergic neurons may shift toward an increased presence of 5-HT_1A_R/5-HT_1A_R homodimers. This shift would result in inhibition of 5-HT release due to the functional properties of 5-HT_1A_ autoreceptors [97]. In postsynaptic neurons, an increase in 5-HT1AR/5-HT7R heterodimers has been proposed, which could indicate greater excitability of serotonergic, glutamatergic, and dopaminergic neurons [97].

Consistent with this hypothesis, previous studies have shown that intermittent chronic stress in rodents results in decreased 5-HT levels in the orbitofrontal cortex [100]. Similar studies have also reported depressive behaviors and cognitive impairment, both of which were reversed after the administration of 5-HT_1A_R agonists [100,101].

Based on these findings, it is also plausible to propose an increase in postsynaptic 5-HT_1A_ homodimer density, which could lead to a decrease in the release of 5-HT, DA, Glu, and other neurotransmitters.

However, a significant challenge remains in explaining the procognitive effects observed with various serotonergic drugs targeting 5-HT_1A_ and 5-HT_7_ receptors. Further studies are needed to understand the dynamics of 5-HT_1A_R and 5-HT_7_R homo- and heterodimerization in both pre- and postsynaptic regions, as well as the factors, mechanisms, and consequences associated with receptor oligomerization in different brain areas involved in memory formation and consolidation.

In line with the above, the procognitive effect observed in most of the reviewed studies following the administration of 5-HT_1A_R agonists, compared to antagonists (Table 2 and Table 3), aligns with previous evidence linking procognitive effects to the activation of the 5-HT_1A_ autoreceptor. This activation may produce opposing effects on 5-HT levels: on one hand, reducing exocytosis, while on the other, potentially inhibiting SERT, thereby prolonging 5-HT availability in the synaptic cleft. This prolonged presence of 5-HT could enhance its interaction with the 5-HT_1A_ heteroreceptor or other receptors, such as 5-HT_7_, which have been associated with increased Glu and DA release following the activation of 5-HT_1A_ heteroreceptors and 5-HT_7_R in pyramidal neurons [94].

Similarly, this explanation aligns with the contrasting effects observed in previous studies on 5-HT_1A_R antagonist administration. It is important to highlight a proposal from another study suggesting that 5-HT_1A_R antagonists, after blocking the 5-HT_1A_ heteroreceptor, could increase postsynaptic Glu release either directly or indirectly. This may occur because blocking 5-HT_1A_R could enhance the function of 5-HT_7_R in pyramidal neurons [94].

**Figure 3:**
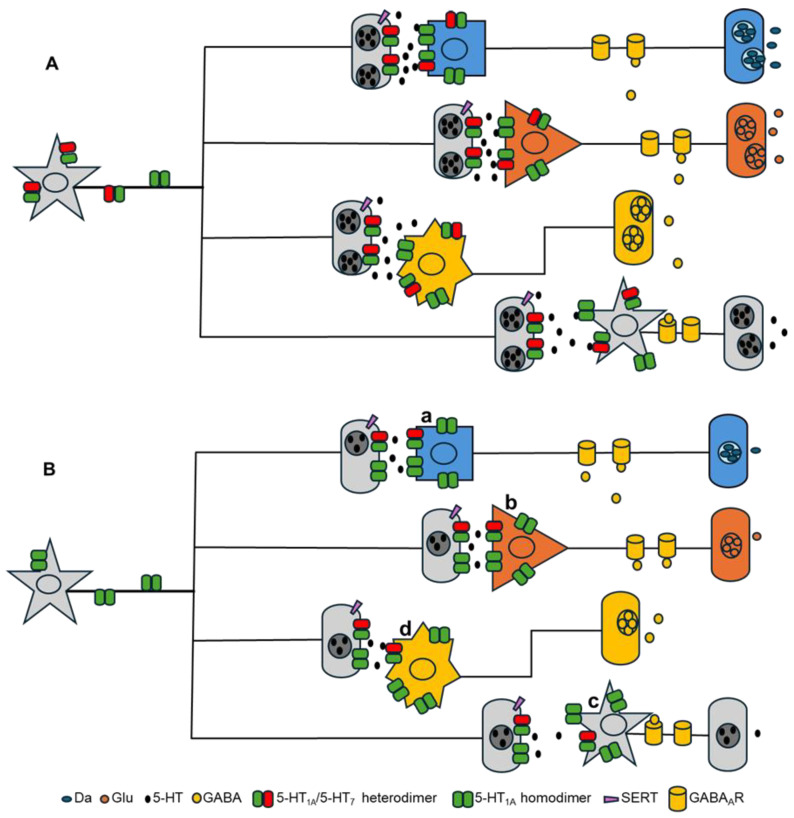
Hypothetical model illustrating the influence of oligomerization on cognitive impairment. (**A**) Under physiological conditions (top), a balance is observed in the postsynapse between the expression of 5-HT_1A_/5-HT_7_ heterodimers and 5-HT_1A_ homodimers, maintaining the postsynaptic release of neurotransmitters, including dopamine (DA), glutamate (Glu), gamma-aminobutyric acid (GABA), and serotonin (5-HT). (**B**) In cognitive impairment, an increase in 5-HT_1A_ homodimer expression compared to 5-HT_1A_/5-HT_7_ heterodimers may occur at the postsynaptic level, leading to a reduction in the exocytosis of DA, Glu, and 5-HT. The postsynaptic localization of the 5-HT_7_ receptor (a, b, c, and d) indicates potential sites of action for serotonergic 5-HT_7_ drugs, where site d may represent a key regulatory point in GABA release by interneurons.

Regarding the contrasting procognitive effects observed following treatment with 5-HT_7_ agonists and antagonists, it is necessary to consider the greater complexity of the system due to multiple interacting factors, including the functional differentiation of 5-HT_7_R depending on its localization. For example, the effect of 5-HT_7_R agonists in postsynaptic cholinergic, glutamatergic, and serotonergic neurons (Figure 3B, sites **a**, **b**, and **c**) could lead to increased exocytosis of ACh, Glu, and DA, thereby enhancing neuronal plasticity. In contrast, an opposing effect could be observed when the same receptor is stimulated in GABAergic interneurons (Figure 3B, site d), where activation would increase GABA release, exerting an inhibitory effect on excitatory systems.

At this same site (**d**), 5-HT_7_ antagonists may act by reducing GABA release, leading to the disinhibition of excitatory systems, which could promote neuronal plasticity. Another possible mechanism of 5-HT_7_ antagonists is their presynaptic action, where they may inhibit SERT [95], thereby increasing serotonergic activity on postsynaptic neurons. These theoretical proposals align with the reported antidepressant and procognitive effects of both 8-OH-DPAT and lurasidone—the former acting as an agonist of both 5-HT_1A_R and 5-HT_7_R, and the latter functioning as a 5-HT_1A_R agonist and 5-HT_7_R antagonist [89,102].

However, given the contradictory effects reported with 5-HT_7_R antagonist administration on cognitive impairment [103] (Table 5), further preclinical research is essential before considering the clinical application of these drugs. Further studies are needed to better establish their potential therapeutic benefits.

It is important to highlight that multiple factors may contribute to the variability in the effects of 5-HT_1A_ and 5-HT_7_ serotonergic drugs on memory. During learning, 5-HT_1A_R synthesis levels have been found to increase, whereas during memory consolidation, a decrease in *5-HT_1A_R mRNA* and an increase in 5-HT_7_R expression have been observed [104]. Additionally, differences in the cognitive paradigms used in studies and the well-recognized biological variability between mice and rats must also be considered [72,80,93].

On the other hand, it is also important to consider a previous narrative review conducted a decade ago, which addressed a similar topic by analyzing the roles of 5-HT_1_AR and 5-HT_7_R in memory separately [94]. In contrast to the present review, it also explored the phenomenon of dimerization and the interaction with other neurotransmitter systems involved in memory.

Nevertheless, it remains essential to continue investigating both the factors that promote increased oligomerization, including homo and heterodimerization of these receptors, and to further explore the intracellular signaling consequences of these coexpressions. Studies using cell cultures or electrophysiological techniques may provide deeper insights into these mechanisms.

## 5. Conclusions

5-HT_1A_R and 5-HT_7_R can be coexpressed in brain regions associated with higher-order functions such as learning and memory, allowing them to critically and specifically regulate these processes. However, since multiple mechanisms contribute to learning and memory, future studies will be necessary to investigate the precise role of these receptors in memory encoding, consolidation, expression, and retention. Their localization in serotonergic, glutamatergic, GABAergic, and dopaminergic neurons plays a major role in this regulation and may help explain the paradoxical effects induced by specific serotonergic drugs (agonists and antagonists).

Furthermore, the formation of 5-HT_1A_R and 5-HT_7_R homo and heterodimers, as well as differences in their relative concentrations in presynaptic and postsynaptic serotonergic neurons, may be critically involved in both the onset and response to treatment of psychiatric disorders such as depression and anxiety. These factors highlight the need for continued research into the relationship between 5-HT_1A_R, 5-HT_7_R, and cognitive impairment using diverse approaches, including gene silencing, electrophysiology, and cell culture studies.

## Data Availability

Not applicable.

## Abbreviations

The following abbreviations are used in this manuscript:

AC	adenylyl cyclase
ACh	Acetylcholine
AS-19	(2S)-N,N-Dimethyl-5-(1,3,5-trimethylpyrazol-4-yl)-1,2,3,4-tetrahydronaphthalen-2-amine
5-HT1AR	5-HT1A receptor
5-HT_7_R	5-HT_7_ receptor Linear dichroism
8-OH-DPAT	8-Hydroxy-2-(di-n-propylamino)tetralin
CM	Carousel Maze
CNC	central nervous system
CS-US,	Conditioned Stimulus–Unconditioned Stimulus
DA	dopamine
DRN	dorsal raphe nuclei
GABA	gamma-aminobutyric acid
Glu	glutamate
GPCR	G protein-coupled receptor
HC	hippocampus
LP-211	N-[(4-cyanophenyl)methyl]-6-[4-(2-phenylphenyl)piperazin-1-yl]hexanamide
NAD-299	(3R)-3-[di(cyclobutyl)amino]-8-fluoro-3,4-dihydro-2H-chromene-5-carboxamide
NORT	Novel Object Recognition Test
OPS	Object Pattern Separation
PA	tested in the Passive Avoidance Task
PCP	phencyclidine
PFC	prefrontal cortex
PLC	phospholipase C
SB-269970	(3-[(2R-2-[2-(4-methylpiperidin-1-yl)ethyl]pyrrolidin-1-yl]sulfonylphenol
SERT	serotonin transporter
STZ	streptozotocin
SYRCLE	Systematic Review Centre for Laboratory Animal Experimentation
WAY-100635	N-[2-[4-(2-methoxyphenyl)piperazin-1-yl]ethyl]-N-pyridin-2-ylcyclohexanecarboxamide
